# Genomic Study of Chromosomally and Plasmid-Mediated Multidrug Resistance and Virulence Determinants in Klebsiella Pneumoniae Isolates Obtained from a Tertiary Hospital in Al-Kharj, KSA

**DOI:** 10.3390/antibiotics11111564

**Published:** 2022-11-06

**Authors:** Ehssan Moglad, Nuor Alanazi, Hisham N. Altayb

**Affiliations:** 1 Department of Pharmaceutics, College of Pharmacy, Prince Sattam bin Abdulaziz University, P.O. Box 173, Alkharj 11942, Saudi Arabia; 2 Department of Biochemistry, Faculty of Science, King Abdulaziz University, Jeddah 21589, Saudi Arabia; 3 Centre for Artificial Intelligence in Precision Medicine, King Abdulaziz University, Jeddah 21589, Saudi Arabia

**Keywords:** *K. pneumoniae*, de novo assembly, virulence factors, NGS, antibiotic resistance genes, Saudi Arabia

## Abstract

*Klebsiella pneumoniae* is an emergent pathogen causing respiratory tract, bloodstream, and urinary tract infections in humans. This study defines the genomic sequence data, genotypic and phenotypic characterization of *K. pneumoniae* clinically isolated from Al-Kharj, KSA. Whole-genome analysis of four *K. pneumoniae* strains was performed, including de novo assembly, functional annotation, whole-genome-phylogenetic analysis, antibiotic-resistant gene identification, prophage regions, virulent factor, and pan-genome analysis. The results showed that K6 and K7 strains were MDR and ESBL producers, K16 was an ESBL producer, and K8 was sensitive to all tested drugs except ampicillin. K6 and K7 were identified with sequence type (ST) 23, while K16 and K8 were identified with STs 353 and 592, respectively. K6 and K7 were identified with the K1 (wzi1 genotype) capsule and O1 serotype, while K8 was identified with the K57 (wzi206 genotype) capsule and O3b. K6 isolates harbored 10 antimicrobial resistance genes (ARGs) associated with four different plasmids; the chloramphenicol acetyltransferase (*catB3*), *bla*_OXA-1_ and *aac(6′)-Ib-cr* genes were detected in plasmid *p*B-8922_OXA-48. K6 and K7 also carried a similar gene cassette in plasmid *p*C1K6P0122-2; the gene cassettes were the trimethoprim-resistant gene (*dfrA14*), integron integrase (*IntI1*), insertion sequence (IS1), transposase protein, and replication initiation protein (RepE). Two hypervirulent plasmids were reported in isolates K6 and K7 that carried synthesis genes (*iucA*, *iucB*, *iucC*, *iucD*, and *iutA*) and iron siderophore genes (*iroB*, *iroC*, *iroD*, and *iroN*). The presence of these plasmids in high-risk clones suggests their dissemination in our region, which represents a serious health problem.

## 1. Introduction

The genus Klebsiella is a Gram-negative bacteria that belongs to the family Enterobacteriaceae, which was discovered by the German microbiologist Edwin Klebs. It is a facultative anaerobe and a non-motile bacillus [1]. *Klebsiella pneumoniae* (KP) causes different types of infections: pneumonia, bacteremia, wound infections, and urinary tract infections. It is an opportunistic pathogen, commonly accompanied by hospital- and community-acquired infections [2,3]. The infections are recurrently severe, particularly affecting patients with a suppressed immune system and neonates [4]. KP is commonly multidrug-resistant (MDR) and is generally recognized to be the main source of antimicrobial resistance genes that can be transferred to other Gram-negative pathogens. According to the Centers for Disease Control and Prevention (CDC), KP was recently classified as an urgent threat to human health [5]. In addition, antibiotic resistance (AR) is currently considered one of the most serious global public health threats, with the possibility to become more challenging by 2020 [5], because of globalization, health system capacity, social, environmental, and demographic changes [6,7]. Of the MDR pathogens, the ESKAPE pathogens (*Enterococcus faecium*, *Staphylococcus aureus*, *Klebsiella pneumoniae*, *Acinetobacter baumanii*, *Pseudomonas aeruginosa* and *Enterobacter* sp.) are described as the most threatening pathogens, and KP is one of them [6].

Moreover, plasmids play a critical role as mobile genetic elements, which can transfer the resistance genes and virulence factors between different bacterial species through mobilization or conjugation [8]. Plasmids also can bring integrative conjugative elements (ICEs), which are mobile genetic elements able to both integrate bacterial chromosomes by site-specific recombination or exist as autonomous plasmid-like conjugative elements [8,9]. ICEs are also known as conjugative transposons that are crucial for horizontal gene transfer between cells, and they can carry insertion sequences and/or transposons, integrases, and the relaxase enzyme, which is critical for conjugation. Antimicrobial resistance (AMR) is continuously evolving and the genes are transferred horizontally through plasmids [10,11].

Currently, there are many different types of plasmids in Enterobacteriaceae; the most commonly described ones are IncF, IncI, IncA/C, IncL (previously designated IncL/M), IncN and IncH [8]. Plasmid-mediated extended-spectrum beta lactamases (ESBLs) are the most prevalent enzymes conferring resistance to most beta-lactam antibiotics [12,13]. Enzymes hydrolyze aminoglycosides and genes encoding for resistance to quinolones, and sulphonamides are often co-transferred through transposons located on a plasmid. In fact, the association of a different range of antibiotic resistance gene classes depends on the plasmid types; for instance, IncF carries a wide variety of gene classes, while IncI plasmids are mainly associated with ESBLs. Some plasmids even have a strong correlation with specific genes, such as IncL/M with *bla*_OXA-48_, or IncK plasmids with *bla*_CMY-2_ or *bla*_CTX-M-14_. KP strains generally harbor more than one plasmid, including the small high-copy number and low-copy number plasmids that are usually large [9].

It has been reported that the KP population is remarkably diverse, containing 100s of independent phylogenetic lineages that vary from each other by ~0.5% nucleotide divergence [14]. Most MDR hospital outbreaks are due to a small subset of KP clones with a high incidence of acquired antimicrobial resistance (AMR) genes, whereas most community-acquired infections are because of hyper-virulent clones which rarely contain acquired AMR genes but have a great occurrence of key virulence loci [15,16]. KP has evolved and become resistant to third-generation cephalosporins and carbapenems, which has led to narrow treatment options for MDR KP, and in some settings, entirely removed [17]. However, there is a continuous surge in the prevalence of MDR KP strains; hypermucoviscous and hypervirulent KP strains have also been acknowledged and described [18].

In this study, KP was selected due to the high prevalence of reported KP infections in Saudi Arabia with high drug resistance levels; specifically, a high resistance rate to carbapenems, which are the first choice of treatment in local guidelines (38.4% for imipenem and 46.1% for meropenem), 40.7% for colistin and 53.3% for tigecycline, which are generally used as alternative selections of treatment [19]. Besides, KP might create a new clinical disaster if cumulative resistance—for instance, MDR, carbapenemase production, and hypervirulence such as hypermucoviscosity—feature [20,21,22]. The general knowledge about the overall genetic diversity of KP strains in Al-Kharj is limited. Therefore, this comparative genomic study aims to understand the genomic characteristics and virulence profiles of the four different KP strains, and provide deep insights into the antimicrobial resistance genes, virulence, and the persistence of progressively essential global pathogens.

## 2. Results

### 2.1. Phenotypic Characterization of the Isolate

The clinical isolates were obtained from a tertiary hospital in Al-Kharj. The origin of the isolates was from vaginal swabs, blood, and urine. The isolates were identified as KP using the standard identification procedure. According to the CLSI breakpoints, the isolates were classified as MDR when showing resistance to at least one drug in three different categories (ampicillin–sulbactam, aztreonam, ceftazidime, cefotaxime, trimethoprim–sulfamethoxazole), while the isolates were identified as extended-spectrum beta lactamases (ESBLs) when they revealed resistance to ceftazidime and cefotaxime.

KP strains 6 and 7 were MDR and ESBLs, strain 16 was an ESBL, and strain 8 was sensitive to all tested drugs except ampicillin (Table 1).

### 2.2. Genome Characterization and Typing

The total genome for the *K. pneumoniae* isolates (K6, K7, K8 and K16) were assembled into 5.39, 5.44, 5.38 and 5.48 Mb, respectively, with an average contig length range of 42,763–96,382, while N50 was 203,642. The number of contigs was ≤126 for all isolates. The protein coding sequences (CDS) of the isolates were 5215, 5284, 5374 and 5417 for the isolates K6, K7, K8 and K16, respectively. PubMLST database was used for the identification of assembled genomes at the species level and displayed 100% identity with *K. pneumoniae*. Kp 6 and Kp7 were identified with sequence types (STs) 23, while Kp 16 and Kp8 were identified with STs 353 and 592, respectively. The global platform for genomic surveillance, Pathogenwatch, was used for the prediction of the capsule (K) and O serotypes; K6 and K7 were identified with the K1 (wzi1 genotype) capsule and O1 serotype, while K8 was identified with K57 (wzi206 genotype) capsule and O3b (Table 2).

### 2.3. Plasmids and Chromosomally Mediated Mobilome

Plasmids generated from plasmidSPAdes and the assembled contigs were screened for the presence of ARGs, virulence genes (VGs), insertion sequences and integrons.

**Figure 1:**
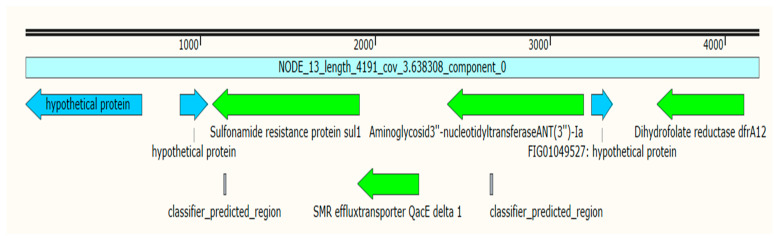
A cluster of antimicrobial resistance genes in the plasmid *p*CEX23 of isolate K6. The green colors represent the sulphonamide-resistant gene (*sul2*), disinfecting agents and antiseptic-resistant gene (*qacE*), trimethoprim-resistant gene (*dfrA12*), and the aminoglycoside-resistant gene (*aadA2*).

**Figure 2:**
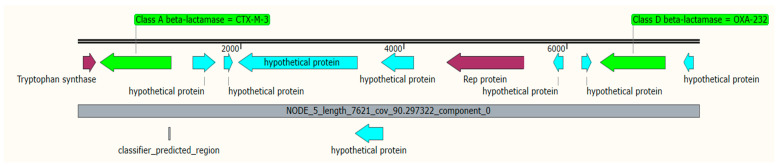
A cluster of antimicrobial resistance genes in the plasmid *p*ColKP3 of isolate K6. The green colors show the *bla*_CTX-M-3_ and *bla*_OXA-232_, while the replication initiation gene is shown in violet color.

**Figure 3:**
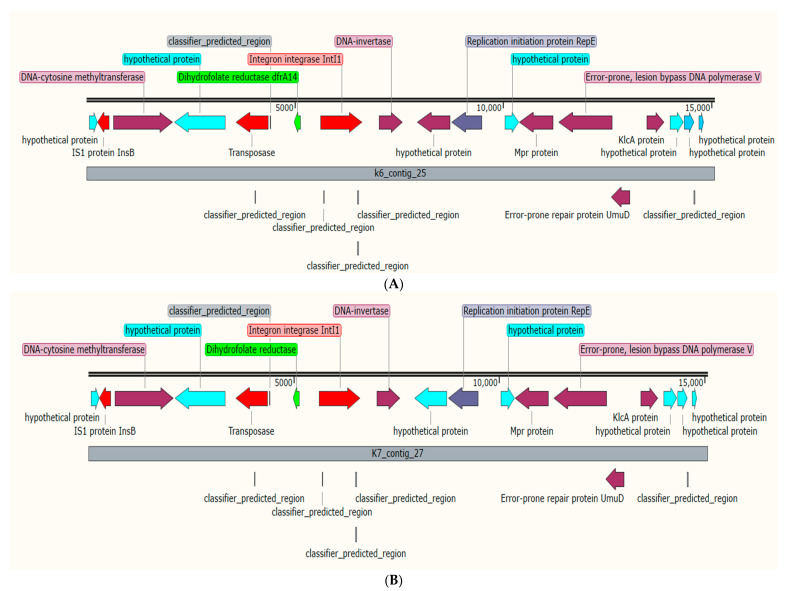
Gene cassettes in plasmid *p*C1K6P0122-2 of isolates K6 (**A**) and K7 (**B**); the gene cassettes were the trimethoprim-resistant gene *dfrA14* (green), integron integrase (*IntI1*) (red), insertion sequence (IS1) (red), transposase protein (red), and replication initiation protein (RepE) (grey).

K6 isolates harbored 10 ARGs associated with four different plasmids; the chloramphenicol acetyltransferase (*catB3*), *bla*_OXA-_1 and *aac(6′)-Ib-cr* genes were detected in plasmid *p*B-8922_OXA-48 (Table 3). Mobile gene cassettes consisting of the sulfonamide-resistant gene (*sul2*), disinfecting agents and antiseptics-resistant gene (*qacE*), trimethoprim-resistant gene (*dfrA12*), and the aminoglycoside-resistant gene (*aadA2*), were detected in plasmid *p*CEX23 and were associated with three integron-related recombination sites (*attC*) as shown in Table 4 and Figure 1. Additionally, isolate K6 harbored the plasmid *p*ColKP3 that carries the extended-spectrum beta-lactamases genes (*bla*_CTX-M-3_ and *bla*_OXA-232_) and the replication initiator protein (Rep) (Figure 2). Both isolates K6 and K7 carried a similar gene cassette in plasmid *p*C1K6P0122-2; the gene cassettes were the trimethoprim-resistant gene (*dfrA14*), integron integrase (*IntI1*), insertion sequence (IS1), transposase protein, and replication initiation protein (RepE) (Figure 3). Additionally, isolate K7 harbored the plasmid *p*1527854_3 which carries the *bla*_CTX-M-3._ Isolate K8 was detected with only one AMR plasmid (*p*MBRCAV1205) that carried the quinolone resistance determinants (*qnrB4* and *qnrB55*), while isolate K16 was detected with plasmid *p*K92-qnrS, which carried a cluster of *qnrS1*, *aph(3″)-Ib*, *sul2*, *aph(6)-Id*, and one insertion sequence (ISKpn19). Another drug-resistant plasmid (*p*IPCEC48_1) was detected with *bla*_CTX-M-15_ (Table 3).

The screening of chromosomally mediated genes revealed the common presence of the fosfomycin resistance (*fosA*) and the multidrug resistance genes (*OqxB* and *OqxA*) in all the isolates (Supplementary File S1, Table S1). The *SHV* ESBLs genes were detected in isolates K6 and K7 (*SHV-190*), K8 (*SHV-26*) and K16 (*SHV-11*), while the gene causing resistance to tetracycline *tet(A)* was detected in isolate K6.

**Figure 4:**
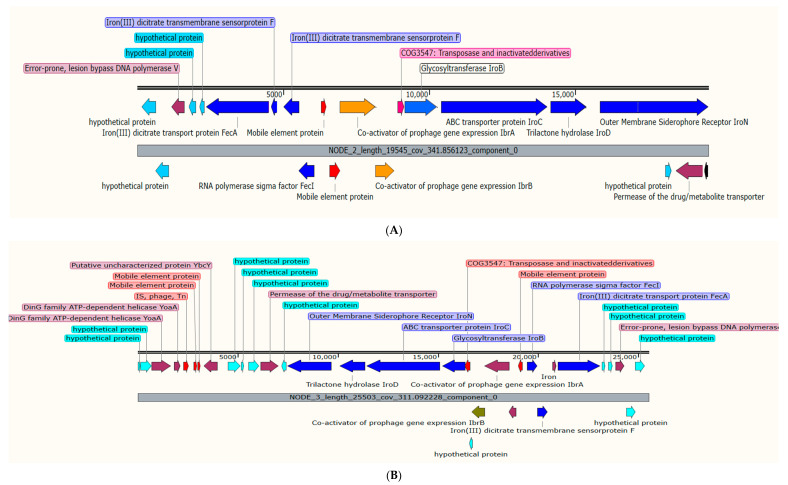
A cluster of enterobactin siderophore receptor genes (*iroB*, *iroC*, *iroD*, and *iroN*) (in blue colors) in the plasmid *p*F1K6P0037-1 of both isolates K6 (**A**) and K7 (**B**). The associated mobile genetic elements are shown in red colors.

**Figure 5:**
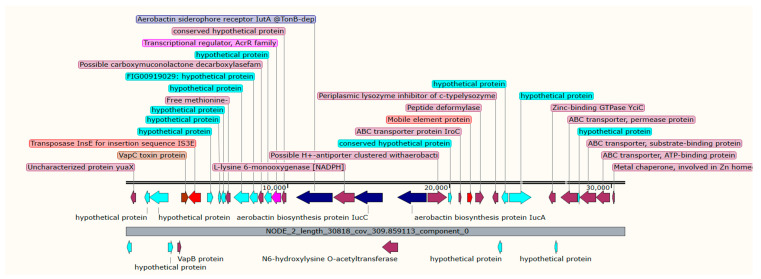
A cluster of plasmid-mediated aerobactin synthesis genes (*iucA*, *iucB*, *iucC*, *iucD*, and *iutA*) (blue), IS3E, mobile element (red), VapC toxin protein (brown), VapB protein and transcriptional regulator (*AcrR*) (light brown), observed in the plasmid *p*EC422_4 of isolate K7.

Investigation of plasmids and chromosomally mediated virulomes revealed the presence of enterobactin siderophore receptor genes (*iroB*, *iroC*, *iroD*, and *iroN*) and two mobile elements in a plasmid (*p*F1K6P0037-1) in both K6 and K7 isolates (Figure 4). The aerobactin synthesis genes (*iucA*, *iucB*, *iucC*, *iucD*, and *iutA*), IS3E, mobile element, VapC toxin protein, VapB protein, and transcriptional regulator (*AcrR*) were observed in the plasmid *p*EC422_4 of isolate K7 (Figure 5). K8 isolate harbored two virulent plasmids: the *p*19-Pyelo_1 (carrying the fimbrial adhesion (*afaD*) and the enterotoxin (*senB*) genes) and *p*YJ6-NDM5, carrying a gene that encodes for the outer membrane proteins—transfer protein (traT) (Tables S1 and S2).

Three virulence factors were identified clustered in the chromosome of K6 and K7 isolates; the iron-regulation gene (*irp2*), the colibactin hybrid non-ribosomal peptide synthetase/type I polyketide synthase encoding gene (*ClbB*) and the siderophore receptor (*fyuA*). Isolate K7 was detected with ABC transporter protein (MchF) and integrated mobile genomic island E492 (GIE492) in a contig that carried *bla*_SHV-190_. Isolate K16 was identified with chromosomally mediated *irp2*, *fyuA*, and tellurium ion resistance protein (*terC*).

The Plasmid Finder tool revealed the existence of three plasmid replicons (IncN, IncFIB(K), and IncHI1B) in the K6 and K7 strains, one plasmid (IncFIB(K)) in K 16, and three plasmids (IncHI1B, IncFIB(K) and IncFIB(AP001918)) in K8 (Tables S3 and S4). The virulence finder revealed that the presence of Yersiniabactin (ybt 1; ICEKp1-47-1LV, ybt 10, ICEKp4, 377 2LV) was found in all strains except K8 (Supplementary Table S5).

### 2.4. Phylogenetic Analysis

**Figure 6:**
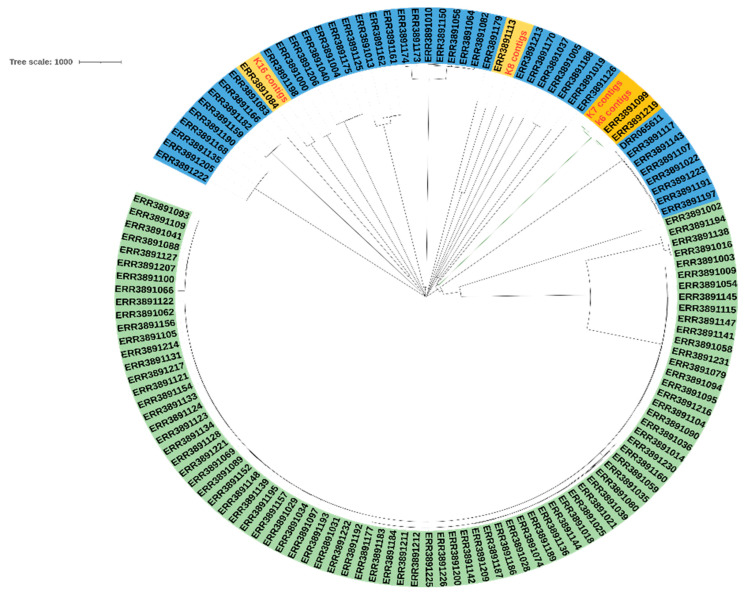
Phylogenetic analysis of our isolates (in red colors) compared to the most similar strains of *K. pneumoniae* isolated from Saudi Arabia. The most similar isolates are highlighted in yellow.

To see the relationship between KP strains, SNP-based phylogenetic analysis was performed after the alignment of the core genome. Isolates K6 and K7 were clustered with two pathogenic strains (ERR3891219, and ERR3891099) of *K. pneumoniae* isolated from human samples at King Abdullah University of Science and Technology, KSA in 2018. While the K8 and K16 were clustered in a different clade containing KP strains from a different region in Saudi Arabia, K8 clustered with ERR3891113 and K8 clustered with ERR3891084 as shown in Figure 6. The metadata of the reference strains used for comparison can be found in Supplementary File S2.

## 3. Discussion

*Klebsiella pneumoniae* is commonly multidrug-resistant (MDR) and is generally recognized to be the main source of antimicrobial resistance genes that can be transferred to other Gram-negative pathogens [23]. According to the Centers for Disease Control and Prevention (CDC), KP was recently classified as an urgent threat to human health [5]. As the platforms for the acquisition and subsequent spread of drug-resistance and virulence genes, plasmids play a crucial role [23,24]. In this study, different plasmids were reported to have drug-resistance and virulence genes, and the ESBL-resistance genes were reported in different multidrug resistance plasmids in isolate K6. The chloramphenicol acetyltransferase (*catB3*), *bla*_OXA-1,_ and *aac(6′)-Ib-cr* genes were detected in plasmid *p*B-8922_OXA-48, and the ESBLs genes (*bla*_CTX-M-3_ and *bla*_OXA-232_) were reported in plasmid *p*ColKP3. The presence of ESBLs genes (*bla*_OXA-1_, *bla*_CTX-M-3_ and *bla*_OXA-232_) in the K6 isolate is consistent with the phenotypic finding in which the isolate was highly resistant to third- and fourth-generations of cephalosporins. Consistent with our findings, Doumith et al. [25] recently reported the presence of the plasmid *p*ColKP3 carrying *bla*_OXA-232_ in *Pseudomonas aeruginosa* clinical isolates from Saudi Arabia, which may indicate the exchange of plasmids among different microbial strains and the circulation of such plasmids in our region. ARGs residing in extrachromosomal plasmid DNA tend to disseminate more into different niches, and the replication initiator protein (Rep) is required for the replication initiation of this plasmid [26]. In this study, a multidrug-resistant plasmid was detected in K6 isolates which harbored extended-spectrum beta-lactamases genes (*bla*_CTX-M-3_), carbapenem-hydrolyzing oxacillinase (*bla*_OXA-232_) [27] and the Rep protein. Since its first description in France from patients returning from India, the blaOXA-232 enzyme has also been described in Singapore, India, Malaysia, the United States, Korea and Tunisia [27]. The emergence of *OXA-232*-producing hypervirulent *K*. *pneumoniae* ST23 has been reported recently in India from a neonate with sepsis [28]. Here, in Saudi Arabia, this is probably the first report of ST23 OXA-232-producing *K. pneumoniae*. The presence of these genes in plasmids could represent a serious health problem due to their easy transmission to different bacterial strains.

Interestingly, in isolate K6, we reported gene cassettes consisting of the sulfonamide-resistant gene (*sul2*), disinfecting agents and antiseptics-resistant gene (*qacE*), trimethoprim resistant gene (*dfrA12*), and the aminoglycoside resistant gene (*aadA2*), in plasmid *p*CEX23 and associated with three integron-related recombination sites (*attC*). In isolates K6 and K7, we reported similar gene cassettes consisting of integron integrase (*IntI1*), trimethoprim-resistant gene (*dfrA14*), insertion sequence (IS), transposase protein, and replication initiation protein (RepE) in plasmid *p*C1K6P0122-2 and lacking the integron-related recombination sites (*attC*). Despite lacking these self-mobility components, integrons encoded by plasmids have the ability to capture genomic structures, express gene cassettes, and mediate their own mobility [29]. The presence of these gene cassettes in plasmids with IS, integrons, or integron-related recombination sites will facilitate the mechanisms of integration or excision of these gene cassettes, which could spread drug-resistant genes among Gram-negative bacteria, thus promoting multidrug resistance in clinical bacterial strains [30,31].

K6 and K7 isolates belong to K1 capsule type and sequence type 23, which are traditionally known as hypervirulent and drug-susceptible [32]; unfortunately, here we reported these isolates with multidrug phenomena and carrying different virulent determinants. Virulome prediction revealed the presence of the enterobactin siderophore iroA locus (that encodes *iroB*, *iroC*, *iroD*, and *iroN*) and two mobile elements in a plasmid (*p*F1K6P0037-1) in both K6 and K7 isolates. These enterobactin siderophores are found in pathogenic Gram-negative bacteria including *K. pneumoniae*, associated with iron acquisition [33], which is essential for bacterial survival and virulence [34]. Plasmid-mediated iron uptake system is rare. Two common plasmid types (ColV and pJM1) have been reported to have iron determinants [34]. Here, we reported for the first time the presence of iroA locus in a plasmid which showed 100% of *p*F1K6P0037-1 of *K. pneumoniae* (accession CP052182.1) in both the K6 and K7 isolates, suggesting the possibility of transmission of this plasmid between the isolates. Additionally, K7 possessed plasmid-mediated gene cassettes carried on a hypervirulent plasmid (*p*EC422_4), which consist of the aerobactin synthesis genes (*iucA*, *iucB*, *iucC*, *iucD*, and *iutA*), IS3E, mobile element, VapC, VapB protein, and transcriptional regulator (*AcrR*); inconsistent with other studies, plasmid-mediated aerobactin genes have been reported widely in *K. pneumoniae*, most commonly in hypervirulent strains [35]. The toxin–antitoxin (TA) system (VapC, VapB) protects the cells from the toxin’s activity and is essential for the survival of pathogenic bacteria [36]. The VapBC TA system is shown to have function on the maintenance of the *Shigella sonnei* virulence plasmid [37] and the MDR genomic island 1 of *Salmonella enterica* [38]. The VapB antitoxin attaches to the VapC toxin and inhibits its activity by cleavage of tRNA^fMet^, preventing the start of translation [37]. Here, all the isolates possessed this system, reflecting its possible essential role in bacterial pathogenicity.

## 4. Methods

### 4.1. Bacterial Isolate and Identification

Four clinical isolates were collected from a tertiary hospital, Al Kharj, KSA, in January 2021. These were isolated from different clinical samples: blood, vaginal swabs and urine. Identification of isolates was performed using Gram stain and automated Microscan Walkaway 96 Plus system following the manufacturer’s instructions.

### 4.2. Antibiotic Susceptibility Testing and Minimum Inhibitory Concentration (MIC)

Susceptibility of all isolates to antibiotics was determined using Microscan negative BP combo panel in automated Microscan Walkaway 96 Plus system. This provided KP identification and susceptibility results on one panel, and MIC detection following latest CLSI guidelines [39]. The antibiotics tested were: Amikacin, Amoxicillin-clavulanic acid, Ampicillin- Sulbactam, Ampicillin, Aztreonam, Cefazolin, Cefepime, Cefotaxime, Cefoxitin, Ceftazidime, Cefuroxime, Ciprofloxacin, Colistin, Ertapenem, Gentamycin, Imipenem, Levofloxacin, Meropenem, Moxifloxacin, Nitrofurantion, Norfloxacin, Piperacillin-Tazobactam, Tigecycline, Tobramycin, and trimethoprim-sulfamethoxazole. For quality control, *K. pneumoniae* ATCC 700603 was used.

### 4.3. Genomic Analysis

Genomic DNA was extracted from fresh-grown isolates using the Guanidine hydrochloride protocol; more details about DNA extraction protocol were published in our previous work [40]. Whole genome sequencing was conducted by Novogene Company (China) using Illumina HiSeq 2500 (Illumina, San Diego, CA, USA), generating 2 × 150 bp paired-end reads. The low-quality generated reads were filtered before the analysis, then the quality control of the raw reads was performed. Genomes were assembled and annotated using the Bacterial and Viral Bioinformatics Resource Center (BV-BRC), and genomes were also annotated using the NCBI Prokaryotic Genome Annotation Pipeline (PGAP) [41]. The identification of assemblies in species, strain levels, and sequence types was conducted using MLST 2.0, PubMLST [42]; sequence types (ST) and capsule-type genes were identified using the global platform for genomic surveillance (Pathogenwatch). Plasmids were assembled from the raw data using the plasmidSPAdes tool v3.15.4, applying different k-mer sizes (21, 33, and 55) [43]. Predicted plasmids were identified by the Plasmid Finder 2.1 tool and BLASTn. Antimicrobial resistance mechanisms were predicted from the assembled plasmid and chromosomes via the Comprehensive Antibiotic Resistance Database (CARD, Version 1.1.3). Virulent Factor Database (VFDB, Version: 2016.03) was used for the identification of virulence mechanisms associated with plasmids and chromosomes. Mobile genetic elements were detected using MGE in center for genomic and epidemiology [8]. Gene maps were generated and visualized by the SnapGene Viewer 6.0.2 software.

### 4.4. Phylogenetic Tree Construction

The SNP-based phylogenetic tree was constructed using the Pathogenwatch phylogenetic tool, and the isolates were compared to all *K. pneumoniae* isolates from Saudi Arabia, which is available in the Pathogenwatch database. The generated tree was downloaded as the Newick tree format and visualized by the online Interactive Tree of Life (iTOL v6) tool that is available at Pasteur MLST.

## 5. Conclusions

The current study reported the presence of multidrug-resistant *K. pneumoniae* isolates circulating in our region that carried plasmids conferring resistance to drug classes, including third- and fourth-generation cephalosporins. Different plasmids were documented to harbor drug-resistant gene cassettes, integrons, and integron-associated recognition sites, which promotes their transmission and dissemination in susceptible isolates. Two hypervirulent plasmids were reported in isolates K6 and K7 that carried synthesis genes (*iucA*, *iucB*, *iucC*, *iucD*, and *iutA*) and iron siderophore genes (*iroB*, *iroC*, *iroD*, and *iroN*). The presence of these plasmids in high-risk clones suggests their dissemination in our region, which represents a serious health problem.

## Figures and Tables

**Table 1:** MIC and antimicrobial susceptibility profile for isolated strains of KP.

		MIC = µg/mL			
	Strains	K6 (Vaginal Swab)	K7 (Blood)	K16 (Urine)	K8 (Urine)
Drugs					
Amikacin		≤16 (S)	≤16 (S)	≤16 (S)	≤16 (S)
Amox/Clav		≤8/4 (S)	≤8/4 (S)	≤8/4 (S)	≤8/4 (S)
Amp/Sulbactam		>16/8 (R)	>16/8 (R)	16/8 (I)	≤8/4 (S)
Ampicillin		>16 (R*)	>16 (R*)	>16 (R*)	>16 (R)
Aztreonam		>16	>16	>16	≤4 (S)
Cefazolin		>4 (R*)	>4 (R*)	>4 (R*)	≤2 (S)
Cefepime		>16 (R*)	>16 (R*)	>16 (R*)	≤8 (S)
Cefotaxime		>16	>16	>16	≤1 (S)
Cefoxitin		≤8 (S)	≤8 (S)	≤8 (S)	≤8 (S)
Ceftazidime		8	8	>16	≤1 (S)
Cefuroxime		>16 (R*)	>16 (R*)	>16 (R*)	≤4 (S)
Ciprofloxacin		≤1 (S)	≤1 (S)	≤1 (S)	≤1 (S)
Colistin		≤2	≤2	≤2	≤2 (S)
Ertapenem		≤0.5 (S)	≤0.5 (S)	≤0.5 (S)	≤0.5 (S)
Gentamycin		≤4 (S)	≤4 (S)	≤4 (S)	≤4 (S)
Imipenem		≤1 (S)	≤1 (S)	≤1 (S)	≤1 (S)
Levofloxacin		≤2 (S)	≤2 (S)	≤2 (S)	≤2 (S)
Meropenem		≤1 (S)	≤1 (S)	≤1 (S)	≤1 (S)
Moxifloxacin		≤0.5 (S)	≤0.5 (S)	1	≤0.5 (S)
Nitrofurantion		≤32 (S)	≤32 (S)	≤32 (S)	≤32 (S)
Norfloxacin		≤4 (S)	≤4 (S)	≤4 (S)	≤4 (S)
Pip/Tazo		≤16 (S)	≤16 (S)	≤16 (S)	≤16 (S)
Tigecycline		≤1(S)	≤1 (S)	≤1 (S)	≤1 (S)
Tobramycin		≤4 (S)	≤4 (S)	≤4 (S)	≤4 (S)
Trimeth/Sulfa		>2/38 (R)	>2/38 (R)	≤2/38 (S)	≤2/38 (S)

Key: S = sensitive, R = resistant, R* = predicted resistant interpretation, ESBL = extended-spectrum beta lactamase.

**Table 2:** Genomic features and assembly information.

ID	Genome Length	No. of Contigs	Average Contig Length	N50	L50	GC Content	CDS	tRNA	rRNA	STs	K Locus Type	O Locus Type
K6	5,397,408	56	96,382	44,7087	4	57.4%	5215	68	3	23	K1	O1
K7	5,446,096	62	87,840	44,7087	4	57.4%	5284	68	3	23	K1	O1
K8	5,388,197	126	42,763	203,642	8	57.2%	5374	76	4	592	K57	O3b
K16	5,483,033	69	79,464	357,616	6	57.1%	5417	74	4	353	K110	O3b

**Table 3:** Plasmids associated with antimicrobial-resistant genes (ARGs) and virulence genes (VG) in KP isolates.

	Plasmid	Identity	Accession	ARG				VG				
K6	*p*B-8922_OXA-48	100%	CP094368.2	*catB3*	*bla*_OXA-1_	*aac(6′)-Ib-cr*						
	*p*CEX23	100%	LC556222.1	*qacE*	*sul1*	*dfrA12*	*aadA2*					
	*p*ColKP3	99	CP036331.1	*bla*_CTX-M-3_	*bla*_OXA-232_							
	*p*C16KP0122-2	99.99%	CP052433.1	*dfrA14*								
	*p*F16KP0037-1	100%	CP052182.1					*iroB*	*iroC*	*iroD*	*iroN*	
K7	*p*1527854_3	100%	CP102116.1	*bla*_CTX-M-3_								
	*p*C16KP0122-2	99.99	CP052433.1	*dfrA14*								
	*p*EC422_4	99.65%	CP018958.1					*iucA*	*iucB*	*iucC*	*iucD*	*iutA*
	*p*F16KP0037-1	100%	CP052182.1					*iroB*	*iroC*	*iroD*	*iroN*	
K8	*p*MBRCAV1205	100%	ON911500.1	*qnrB4*	*qnrB55*							
	*p*19-Pyelo_1	93%	CP048854.1					*afaD*	*senB*			
	*p*YJ6-NDM5	100%	AP023236.1					*traT*				
K16	*p*IPCEC48_1	100%	AP026795.1	*bla*_CTX-M-15_								
	*p*K92-qnrS	100%	OL828743.1	*qnrS1*	*aph(3″)-Ib*	*sul2*	*aph(6)-Id*					

**Table 4:** Integron and integron-related recombination sites (*attC*) detected in isolated K6 and K7.

ID_Integron	Element	Pos_Beg	Pos_End	Strand	E Value	Type_Elt	Annotation	Model
Integron 1	NODE_13_	1902	2249	−1	NA	protein	protein	NA
	attc_001	2352	2411	−1	2.70 × 10^−10^	attC	attC	attc_4
	NODE_13_	2413	3192	−1	NA	protein	protein	NA
	attc_002	3208	3267	−1	1.90 × 10^−7^	attC	attC	attc_4
	attc_003	3528	3617	−1	5.20 × 10^−6^	attC	attC	attc_4
	NODE_13	3612	4109	−1	NA	protein	protein	NA
	NODE_23	140	1153	1	9.10 × 10^−27^	protein	intI	Inter section_tyr_intI

## Data Availability

The data for this project was submitted to GenBank under the Bioproject PRJNA767482 and in the additional files. The following Biosamples were provided SAMN31116560, SAMN31116561, SAMN31116562 and SAMN31116563. The samples genome accession number were as follows: K16:JAOTEB000000000, K6: JAOTDY000000000, K7:JAOTDZ000000000 and K8: JAOTEA000000000.

## Supplementary Materials

The following supporting information can be downloaded at: https://www.mdpi.com/article/10.3390/antibiotics11111564/s1, Table S1: Resistance gene using ResFinder. Table S2: Detection of AMR in genome using RGI Resistance Gene Identifier. Table S3: Detection of Plasmid using PlasmidFinder in Center for Genomic and Epidemiology. Table S4: Detection of Plasmid using MobileElementFinder in Center for Genomic and Epidemiology. Table S5: Detection of the Virulence factor in Genome sequences of isolates.
