# Supplementary material for: Genomic Study of Chromosomally and Plasmid-Mediated Multidrug Resistance and Virulence Determinants in Klebsiella Pneumoniae Isolates Obtained from a Tertiary Hospital in Al-Kharj, KSA
Source: Antibiotics (Basel). 2022 Nov 6;11(11):1564. doi: 10.3390/antibiotics11111564 (PMC9686629; doi:10.3390/antibiotics11111564)
Supplement: Supplementary file 1 [file antibiotics-11-01564-s001.zip › Supplementary file.pdf]

Table S1: Resistance gene using ResFinder

	Gene name	Phenotype	Accession	Position in contig	Coverage	Identity
K8	blaSHV-26	ticarcillin+clavulanic acid, piperacillin, amoxicillin+clavulanic acid, ampicillin, amoxicillin, piperacillin+tazobactam, ticarcillin, ampicillin+clavulanic acid	AF227204	1-861	100%	100%
	OqxB	nalidixic acid, ciprofloxacin, chloramphenicol, trimethoprim, benzylkonium chloride, cetylpyridinium chloride	EU370913	1-3153	100%	98.83%
	OqxA	nalidixic acid, ciprofloxacin, chloramphenicol, trimethoprim, benzylkonium chloride, cetylpyridinium chloride	EU370913	1-1176	100%	99.32%
	fosA5	fosfomycin	EU195449	1-420	100%	98.33%
K6	blaCTX-M-3	cefotaxime, ampicillin, amoxicillin, cefepime, ticarcillin, ceftazidime, piperacillin, ceftriaxone, aztreonam	Y10278	1-876	100%	100%
	blaSHV-190	unknown beta-lactam	KP868753	1-861	100%	99.88%
	fosA6	fosfomycin	KU254579	1-420	97.00%	98.33%
	OqxB	trimethoprim, ciprofloxacin, nalidixic acid, benzylkonium chloride, cetylpyridinium chloride, chloramphenicol	EU370913	1-3153	100%	98.76%
	OqxA	trimethoprim, ciprofloxacin, nalidixic acid, benzylkonium chloride, cetylpyridinium chloride, chloramphenicol	EU370913	1-1176	100%	99.15%
K16	blaSHV-85	amoxicillin, piperacillin, ticarcillin, cephalothin, ampicillin	DQ322460	1-861	100%	99.88%
	blaSHV-40	amoxicillin, piperacillin, ceftriaxone, ticarcillin, aztreonam, cefotaxime, ceftazidime, ampicillin, cefepime	AF535128	1-861	100%	99.88%
	blaSHV-79	amoxicillin, piperacillin, ticarcillin, cephalothin, ampicillin	AM176554	1-861	100%	99.88%

	blaSHV-56	amoxicillin, piperacillin, ticarcillin, ampicillin+clavulanic acid, amoxicillin+clavulanic acid, ampicillin, piperacillin+tazobactam, ticarcillin+clavulanic acid	EU586041	1-861	100%	99.88%
	blaSHV-89	amoxicillin, piperacillin, ticarcillin, cephalothin, ampicillin	DQ193536	1-861	100%	99.88%
	qnrS1	ciprofloxacin	AB187515	1-657	100%	100%
	fosA6	fosfomycin	KU254579	1-420	97.00%	99.52%
	OqxB	chloramphenicol, benzylkonium chloride, cetylpyridinium chloride, nalidixic acid, ciprofloxacin, trimethoprim	EU370913	1-3153	100%	99.02%
	aph(3")-Ib	streptomycin	AF321551	1-804	100%	99.88%
	aph(6)-Id	streptomycin	CP000971	7-837	100%	100%
	tet(A)	tetracycline, doxycycline	AJ517790	1-1200	100%	100%
	OqxA	chloramphenicol, benzylkonium chloride, cetylpyridinium chloride, nalidixic acid, ciprofloxacin, trimethoprim	EU370913	1-1176	100%	99.40%
	aph(3')-Ia	ribostamycin, neomycin, kanamycin, lividomycin, paromomycin	V00359	1-816	100%	100%
	sul2	sulfamethoxazole	AY034138	1-816	100%	100%
	blaCTX-M-15	amoxicillin, piperacillin, ceftriaxone, ticarcillin, aztreonam, cefotaxime, ceftazidime, ampicillin, cefepime	AY044436	1-876	100%	100%
K7	blaSHV-190	unknown beta-lactam	KP868753	1-861	100%	99.88%
	fosA6	fosfomycin	KU254579	1-420	97.00%	98.33%
	OqxB	ciprofloxacin, nalidixic acid, chloramphenicol, trimethoprim, benzylkonium chloride, cetylpyridinium chloride	EU370913	1-3153	100%	98.76%
	blaCTX-M-3	aztreonam, cefotaxime, ampicillin, ceftriaxone, ceftazidime, ticarcillin, cefepime, amoxicillin, piperacillin	Y10278	1-876	100%	100%
	OqxA	ciprofloxacin, nalidixic acid, chloramphenicol, trimethoprim,	EU370913	1-1176	100%	99.15%

		benzylkonium chloride, cetylpyridinium chloride				

Table S2: Detection of AMR in genome using RGI Resistance Gene Identifier

ARO term	Drug Class	Resistance mechanism	% identity	% coverage	K16	K6	K7	K8
sul2	sulfonamide antibiotic	antibiotic target replacement	100.0	100.00	√	-	-	-
SHV-11	carbapenem, cephalosporin, penam	antibiotic inactivation	100.0	100.00	√	√	√	-
SHV-26	carbapenem, cephalosporin, penam	antibiotic inactivation	100.0	100.00	-	-	-	√
QnrS1	fluoroquinolone antibiotic	antibiotic target protection	100.0	100.00	√	-	-	-
LptD	carbapenem, peptide antibiotic, aminocoumarin antibiotic, rifamycin antibiotic	antibiotic efflux	100.0	100.00	√	√	√	√
Klebsiella pneumoniae KpnF	macrolide antibiotic, aminoglycoside antibiotic, cephalosporin, tetracycline antibiotic, peptide antibiotic, rifamycin antibiotic, disinfecting agents and antiseptics	antibiotic efflux	100.0	100.00	√	√	√	√
CTX-M-15	cephalosporin, penam	antibiotic inactivation	100.0	100.00	√	-	-	-
Klebsiella pneumoniae KpnE	macrolide antibiotic, aminoglycoside antibiotic, cephalosporin, tetracycline antibiotic, peptide antibiotic, rifamycin antibiotic,	antibiotic efflux	100.0	100.00	-	√	√	-

	disinfecting agents and antiseptics							
oqxA	fluoroquinolone antibiotic, glycylcycline, tetracycline antibiotic, diaminopyrimidine antibiotic, nitrofuran antibiotic	antibiotic efflux	100.0	100.00	-	√	√	-
CTX-M-3	cephalosporin, cephamycin, penam	antibiotic inactivation	100.0	100.00	-	√	√	-
tet(A)	tetracycline antibiotic	antibiotic efflux	99.74	94.10	√	-	-	-
eptB	peptide antibiotic	antibiotic target alteration	99.46	97.04	√	-	√	√
OmpA	monobactam, carbapenem, cephalosporin, cephamycin, penam, penem	reduced permeability to antibiotic	99.45	97.33	√	√	√	√
Klebsiella pneumoniae KpnG	macrolide antibiotic, fluoroquinolone antibiotic, aminoglycoside antibiotic, carbapenem, cephalosporin, penam, peptide antibiotic, penem	antibiotic efflux	99.74	100.00	√	√	√	√
oqxA	fluoroquinolone antibiotic, glycylcycline, tetracycline antibiotic, diaminopyrimidine antibiotic, nitrofuran antibiotic	antibiotic efflux	99.74	100.00	√	-	-	√
Klebsiella pneumoniae OmpK37	monobactam, carbapenem, cephalosporin, cephamycin, penam, penem	reduced permeability to antibiotic	99.47	100.00	√	√	√	-
APH(6)-Id	aminoglycoside antibiotic	antibiotic inactivation	99.28	100.00	√	-	-	-
ArnT	peptide antibiotic	antibiotic target alteration	99.27	100.00	√	√	√	√
APH(3")-Ib	aminoglycoside antibiotic	antibiotic inactivation	99.25	100.00	√	-	-	-
Klebsiella pneumoniae KpnE	macrolide antibiotic, aminoglycoside antibiotic, cephalosporin,	antibiotic efflux	99.17	100.00	√	-	-	√

	tetracycline antibiotic, peptide antibiotic, rifamycin antibiotic, disinfecting agents and antiseptics							
CRP	macrolide antibiotic, fluoroquinolone antibiotic, penam	antibiotic efflux	99.05	100.00	√	√	√	√
FosA6	fosfomycin	antibiotic inactivation	98.56	100.00	√	√	√	-
fosA5	fosfomycin	antibiotic inactivation	99.28	100.00	-	-	-	√
APH(3')-Ia	antibiotic	antibiotic inactivation	98.52	100.00	√	-	-	-
Escherichia coli UhpT with mutation conferring resistance to fosfomycin	fosfomycin	antibiotic target alteration	95.03	100.00	√	-	√	√
Escherichia coli EF-Tu mutants conferring resistance to Pulvomycin	elfamycin antibiotic	antibiotic target alteration	97.97	84.35	-	√	√	-

Table S3: Detection of Plasmid using PlasmidFinder in Center for Genomic and Epidemiology

	Plasmid name	Database	Accession	Position in contig	Coverage	Identity
K8	IncHI1B	Enterobacteriaceae	JN420336	86-655	100%	99.30%
	IncFIB(K)	Enterobacteriaceae	JN233704	300-859	100%	91.25%
	IncFIB(AP001918)	Enterobacteriaceae	AP001918	1-603	88.42%	99.34%
K6	IncN	Enterobacteriaceae	AY046276	39-552	100%	99.81%
	IncFIB(K)	Enterobacteriaceae	JN233704	300-859	100%	91.25%
	IncHI1B	Enterobacteriaceae	JN420336	558-86	82.98%	99.15%
K16	IncFIB(K)	Enterobacteriaceae	JN233704	300-859	100%	91.43%
K7	IncFIB(K)	Enterobacteriaceae	JN233704	300-859	100%	91.25%
	IncN	Enterobacteriaceae	AY046276	39-552	100%	99.81%
	IncHI1B	Enterobacteriaceae	JN420336	558-86	82.98%	99.15%

Table S4: **Detection of Plasmid using MobileElementFinder in Center for Genomic and Epidemiology**

	Plasmid	Identity	Query / Template length	Contig	Position in contig	Not e	Accession number
K16	IncFIB(K)(pCAV1099-114)	100	560 / 560	fig|573.47619.peg.5105 DNA replication protein [Klebsiella pneumoniae K16 | 573.47619]	300..859		CP011596
k7	IncHI1B(pNDM-MAR)	99.15	473 / 570	fig|573.47617.peg.5064 DNA replication protein [Klebsiella pneumoniae K7 | 573.47617]	86..558		JN420336
	IncN	99.81	514 / 514	fig|573.47617.peg.5162 Replication initiation protein RepE [Klebsiella pneumoniae K7 | 573.47617]	39..552		AY046276
	repB	100	560 / 560	fig|573.47617.peg.5111 DNA replication protein [Klebsiella pneumoniae K7 | 573.47617]	300..859	VIR	AP006726
k6	IncHI1B(pNDM-MAR)	99.15	473 / 570	fig|573.47594.peg.4999 DNA replication protein [Klebsiella pneumoniae K6 | 573.47594]	86..558		JN420336
	IncN	99.81	514 / 514	fig|573.47594.peg.5114 Replication initiation protein RepE [Klebsiella pneumoniae K6 | 573.47594]	39..552		AY046276
	repB	100	560 / 560	fig|573.47594.peg.5046 DNA replication protein [Klebsiella pneumoniae K6 | 573.47594]	300..859	VIR	AP006726
k8	IncFIA(HI1)	98.79	247 / 388	fig|573.47618.peg.5077 Replication initiation protein RepE [Klebsiella pneumoniae K8 | 573.47618]	1..247		AF250878
	IncFIB(AP001918)	99.34	603 / 682	fig|573.47618.peg.4850 RepFIB replication protein A [Klebsiella pneumoniae K8 | 573.47618]	1..603		AP001918
	IncHI1B(pNDM-MAR)	99.3	570 / 570	fig|573.47618.peg.4359 DNA replication protein [Klebsiella pneumoniae K8 | 573.47618]	86..655		JN420336
	repB	100	560 / 560	fig|573.47618.peg.4335 DNA replication protein [Klebsiella pneumoniae K8 | 573.47618]	300..859	VIR	AP006726

Table S5: Detection of the Virulence factor in Genome sequences of isolates

	Gene name	VirulenceFinder db	Protien function	Accession	Position in contig	Coverage	Identity
K8	traT	Escherichia coli	Outer membrane protein complement resistance	AAJU02000030	1-732	100%	100%
	iroN	Escherichia coli	Enterobactin siderophore receptor protein	AP006726	1-2175	100%	100%
	terC	Escherichia coli	Tellurium ion resistance protein	UDGH01000006	1-1041	100%	99.81%
	senB	Escherichia coli	Plasmid-encoded enterotoxin	CP000038	1-1176	100%	99.74%
	iutA	Escherichia coli	Ferric aerobactin receptor	UDFN01000030	2202-481	100%	99.59%
	afaD	Escherichia coli	Afimbrial adhesion	LT985252	1-416	99.76 %	99.04%
	iutA	Escherichia coli	Ferric aerobactin receptor	FLWH01000001	1-2190	100%	99.27%
	traT	Escherichia coli	Outer membrane protein complement resistance	FLWH01000012	1-732	100%	98.63%
	iucC	Escherichia coli	Aerobactin synthetase	CYAZ01000069	1-1728	99.14 %	90.69%
K6	iroN	Escherichia coli	Enterobactin siderophore receptor protein	AP006726	1-2175	100%	100%
	fyuA	Escherichia coli	Siderophore receptor	CAY233333	1-2022	100%	100%
	mchF	Escherichia coli	ABC transporter protein McHF	CP003785	2097-25	100%	100%
	irp2	Escherichia coli	High molecular weight protein 2 non-ribosomal peptide synthetase	NZ_NLWN01000039	1-6108	100%	99.56%
	clbB	Escherichia coli	Hybrid non-ribosomal peptide / polyketide megasynthase	AM229678	1-9621	100%	99.98%
	iutA	Escherichia coli	Ferric aerobactin receptor	FLXF01000001	1-2190	100%	99.50%
K16	iutA	Escherichia coli	Ferric aerobactin receptor	FLWH01000001	1-2190	100%	99.86%
	irp2	Escherichia coli	High molecular weight protein 2 non-ribosomal peptide synthetase	NZ_NOSN01000022	1-6108	100%	99.89%
	terC	Escherichia coli	Tellurium ion resistance protein	UGAE01000003	1-1041	100%	99.23%
	fyuA	Escherichia coli	Siderophore receptor	CAY233333	1--2022	100%	100%
K7	irp2	Escherichia coli	High molecular weight protein 2 non-ribosomal peptide synthetase	NZ_NMKL01000024	1-6108	100%	99.56%

	irp2	Escherichia coli	High molecular weight protein 2 non-ribosomal peptide synthetase	NZ_UEMT01000018	1-6108	100%	99.56%
	irp2	Escherichia coli	High molecular weight protein 2 non-ribosomal peptide synthetase	NZ_NLWN01000039	1-6108	100%	99.56%
	iutA	Escherichia coli	Ferric aerobactin receptor	FLXF01000001	1-2190	100%	99.50%
	iutA	Escherichia coli	Ferric aerobactin receptor	UDFN01000030	2202-481	100%	99.65%
	iroN	Escherichia coli	Enterobactin siderophore receptor protein	AP006726	1-2175	100%	100%
	mchF	Escherichia coli	ABC transporter protein MchF	CP003785	2097-25	100%	100%
	clbB	Escherichia coli	Hybrid non-ribosomal peptide / polyketide megasynthase	AM229678	1-9621	100%	99.98%
	fyuA	Escherichia coli	Siderophore receptor	CAY233333	1-2022	100%	100%
	iucC	Escherichia coli	Aerobactin synthetase	CYAZ01000069	1-1728	99.14 %	90.69%
